# Workplace Health Promotion in German Social Firms—Offers, Needs and Challenges from the Perspectives of Employees, Supervisors and Experts

**DOI:** 10.3390/ijerph19020959

**Published:** 2022-01-15

**Authors:** Ann-Christin Kordsmeyer, Ilona Efimov, Julia Christine Lengen, Volker Harth, Stefanie Mache

**Affiliations:** Institute for Occupational and Maritime Medicine (ZfAM), University Medical Center Hamburg-Eppendorf (UKE), 20459 Hamburg, Germany; i.efimov@uke.de (I.E.); j.lengen@uke.de (J.C.L.); harth@uke.de (V.H.); s.mache@uke.de (S.M.)

**Keywords:** health promotion, occupational health, social enterprises, social firms, health behavior

## Abstract

On the general labor market, social firms provide 30–50% of people with different types of disabilities the opportunity to gain employment. However, the topic of workplace health promotion (WHP), needs for improvement and accompanied challenges are neglected in the current research and were the focus of the present study. Therefore, data triangulation was used between July and December 2020 by combining three focus groups with employees (*n* = 14 employees) with 16 interviews with supervisors from several social firms in Northern Germany (e.g., from catering, cleaning or bicycle repair sectors). 17 semi-structured telephone interviews with experts in the field of WHP or social firms were added. All approaches were audio-taped, transcribed and anonymized. To analyze the data, Mayring’s qualitative content analysis was used. The results indicated that several offers for WHP, including sport, nutrition and relaxation, were offered, as well as those on smoking cessation, cooperation with external organizations or training and education offers. Needs for improvement were stated referring to additional sport offers, support for implementing a healthy diet, offers for relaxation, financial incentives or collaborations with external organizations. A low take-up of offers; a lack of resources, structures or management support; compatibility of offers with work time and organization; challenges with available trainings or the consideration of individual needs and capacities were highlighted as challenges. Overall, there is a need for further interventional and longitudinal research on WHP in social firms.

## 1. Introduction

Promoting and maintaining the health of employees is important for companies, not only referring to legal requirements and individual well-being, but also to increase employee motivation and performance as well as employer attractiveness [1]. In 2019, 24.3% of days absent from work were caused by musculoskeletal diseases and 16.8% were caused by mental disorders, being the two highest diagnosis groups [2]. In particular, occupational groups facing high levels of interpersonal interaction were significantly affected by increased sickness absences due to mental disorders (such as health and social services or education [2]). To ensure a sustainable and holistic promotion of the employee health, occupational health management can be defined as the planning, implementation, evaluation and control of health-promoting measures within a company. Such measures include the pillars of occupational health and safety, occupational integration management and workplace health promotion (WHP) [3]. WHP can contribute to keeping the workforce healthy, for instance in terms of diet-related outcomes [4], physical activity [5], mental well-being [6], decreased sickness absences [7] and increased work ability [8]. Associated economic effects also underscore the need to focus on this topic, not only in terms of direct costs of illness (mental disorders account for 44.4 billion euros in 2015 [9]), but also in terms of indirect costs due to production downtime, which amounts to 14.4 billion euros in 2019 in Germany [10]. On a company level, reviews showed a positive return on investment (for every euro invested, 2.70 euros can be saved [11]).

Providing a starting point, the German legislature strengthened health promotion in living environments with the Prevention Act of 2015, including the workplace setting. Unfortunately, people with a disability are not yet sufficiently taken into account in current prevention offers or in the current state of research [12,13,14]. Nevertheless, according to the UN Convention on the Rights of Persons with Disabilities (Article 27), offers for WHP maintaining suitable working conditions should be made available to people with disabilities. Individual studies or interventions show the first efforts towards WHP for people with disabilities in workshop settings, e.g., on mental health [15], health literacy [16,17] and physical activity [18,19]. Providing a more broad national overview, Hublet et al. [20] showed that WHP interventions were implemented in about 65% of settings, such as sheltered and social workshops in Belgium. However, insufficient adaptions to meet the needs of people with disabilities, a lack of data and a lack of transparency were reported [13,20]. Yet, WHP is particularly important for people with disabilities, since they often rate their subjective state of health and psychological well-being lower than people without disabilities or face a higher health burden. In addition, disabled people are often less physically active and in some cases exhibit less favorable health behaviors with respect to their dietary habits or consumption of stimulants [21,22].

In Germany, there are 7.9 million people living with a severe disability. Currently, 57% of people with disabilities between the ages of 15 and 64 are employed or are looking for work [23]. One possibility to gain employment is represented by social firms. Social firms offer people with a severe disability the opportunity of employment on the labor market. The companies must employ at least 30% and up to a maximum of 50% of people with a mental, intellectual, physical, sensory or multiple disabilities, which can serve as a transition between a sheltered workshop and companies on the general labor market (§215, Book Nine of the German Social Code (SGB IX)). Recent legislative changes (§216, Book Nine of the German Social Code (SGB IX)) for this employment possibility took place in 2018 following the obligation to provide offers for WHP, representing a significant difference to other companies on the general labor market. Although an increasing body of literature exists on work accommodations for employees in social firms, scientific studies dealing with the development, implementation and evaluation of WHP are missing [12]. Table 1 summarizes the current research, which serves as the scientific foundation for our study.

### 1.1. Theoretical Framework

As a theoretical framework of the study, the Ottawa Charter was applied, which can be traced back to the first international conference on health promotion in 1986. Following a holistic approach, five action fields for health promotion were described, including health-promoting living environments [24]. The concept of a setting as a social system (e.g., a neighborhood, workplace or educational institution) gained particular relevance within the Ottawa Charter as a response to the limited success of traditional educational activities. As a supplement with a particular focus on the workplace, the Luxembourg Declaration on Workplace Health Promotion in the European Union was added to the theoretical framework of the study [25]. In this context, WHP includes at least two strategies: behavioral-related (individual behavior patterns) and structural-related measures (changing working conditions) from various fields [25]. Since there is a growing body of literature addressing working conditions in social firms [12], also with reference to the German context [26,27], the following study will focus on behavioral-related measures for WHP in the first place. However, it should be noted that past reviews evaluated holistic WHP approaches considering multifactorial interventions on structural and behavioral dimensions as being most effective [28].

### 1.2. Aim of the Study

To gain insight into the topic of WHP for employees and supervisors in social firms, three main research questions were addressed within the present study:

1. How do social firms implement offers for WHP according to employees, supervisors and experts?
2. What needs for improvement on WHP in social firms are stated by employees, supervisors and experts?
3. What challenges play a role when implementing measures for WHP in social firms based on the supervisors’ and experts’ opinions?

## 2. Materials and Methods

### 2.1. Study Design

Employees with disabilities and supervisors of social firms from Northern Germany as well as experts (see definition Section 2.2. Participant Selection) were interviewed about WHP in social firms, needs for improvement and associated challenges during the implementation. Therefore, qualitative data triangulation was applied to tackle the research gap in this occupational setting and gain an in-depth understanding of the different perspectives [29,30]. Qualitative data were collected in parallel between July and December 2020. Here, 16 semi-structured telephone interviews with supervisors, 17 with experts in the field and three focus groups consisting of 3–6 employees from existing teams (14 employees in total) were used. No personal relationship with employees, supervisors or experts existed beforehand. Focus groups with employees were conducted by J.C.L. (M.Sc.) and A.-C.K. (M.A.), both female health scientists. Data analysis for the focus groups was managed by J.C.L. and I.E. (M.Sc.) in collaboration. Semi-structured interviews with supervisors and experts in the field were conducted and analyzed by A.-C.K. All interviewers were employed as research associates and had personal experience in conducting qualitative research.

### 2.2. Participant Selection

Focus group participants and supervisors were recruited via convenience sampling based on regional accessibility in Northern Germany. Beforehand, on-site visits in the workplace were conducted within one workday, where researchers and participants became acquainted with each other, established some trust and highlighted scientific and personal information for the study. In the same vein, the researchers were able to gain an impression of how premises, work activities, equipment and processes were designed as well as breaks, communication and interaction. Afterwards, researchers invited employees to participate in the focus groups and supervisors in the telephone interviews via a request to the social firms’ management. Experts were enrolled via snowball sampling by subsequently asking the participants to name further experts [31]. Based on Schütz [32], experts are characterized by detailed and specialized knowledge regarding a clearly defined field of knowledge. The field can refer not only to scientific knowledge, but includes all forms of special knowledge from society, politics, business or civil society [33]. Meuser and Nagel [34] distinguish between contextual knowledge (comprehensive explicit knowledge about actors, institutions and rules in a field in which the expert himself is not or only marginally active) and operational knowledge (implicit insider knowledge about routines, subjective perceptions and effects in the field under consideration). Often both forms of knowledge are queried in an interview [34]. However, the present research focused primarily on operational knowledge. The applied inclusion criteria for all participants are displayed in Table 2.

Before the focus groups were conducted, no information was available on the number of employees who did not want to participate in the focus groups. This was mainly traced back to the gatekeeper function of supervisors during data collection. The supervisors’ feedback on reasons for employees’ non-participation referred to a lack of motivation or confidence, fears when things were misunderstood or when saying something the wrong way, sickness absence or language barriers. Likewise, two supervisors and one expert ignored recurring invitations for study participation. Reasons were stated for supervisors, including time pressure or challenges due to the COVID-19 pandemic. For experts, no reasons were given why they did not want to participate.

### 2.3. Data Collection

Focus groups within existing teams were evaluated as the most suitable method for depicting different perspectives, experiences and thoughts by strengthening employee confidence and trust in comparison to individual interviews [35]. For employees with disabilities, one focus group was conducted in the social firm and their workplace. The following two focus groups were organized in external conference rooms. Participating employees of each focus group worked together in the same team and social firm. At the start of each focus group, researchers explained the study aim and conversational rules [35]. Postcards with comics on occupational health themes with everyday situations from working life were distributed to stimulate the initial phase of the conversation (e.g., “Pick a card in which you recognize a positive aspect of your work and explain what you like about it”). The comics were provided in agreement with a related WHP project for the workshop setting for methodological use in the focus groups [36]. At the end of each focus group, a short questionnaire was filled out by employees to collect demographic data on gender, age and working experiences. Not only for the moderator’s orientation and focus on the research questions, but also for fostering comparability and reliability, a semi-structured interview guide was developed for the focus groups [35,37]. To ensure comprehensibility of the guide’s questions, plain language was applied in cooperation with a provider of health promotion offers for people with disabilities.

As a supplement, supervisors and experts were interviewed by using telephone interviews when at home or at their workplace, increasing flexibility during the COVID-19 pandemic. Participants of both target groups were asked to seek a quiet place when conducting the interview to avoid any disruptions (one supervisor and one expert were disrupted during an interview at work by colleagues and six experts had technical difficulties during the interviews). When interviewing supervisors, a semi-structured interview guide referring to Witzel’s problem-centered interview technique (PCI) was used, sociodemographic data were collected at the end and postscripts were drafted (main content of the conversation, non-verbal hints and interpretation ideas) [38].

The data collection for the expert interviews followed similar approaches. Given the fact that operational knowledge is composed of a mixture of explicit and implicit knowledge, methodological challenges arise during data collection, which is why semi-structured interview guides and their flexible handling are particularly suitable compared to other approaches. In the course of developing the interview guide for experts, care was taken to attribute thematic competence to the interviewer, since the willingness of experts to share their knowledge depends on how the interviewer’s competence is assessed in terms of the rules, regulations and legal bases [34]. Overall, data collection was conducted in German. All interview guides were tested and optimized beforehand. The interview guide for the focus group was approved with a person working in a comparable setting and those for supervisors and experts with a researcher’s colleague ensuring comprehensibility and practicability. The following topics were included (Table 3):

All participants of the study gave written informed consent after being informed about the study, including its aims, methods, analysis and data protection. Above all, study participation was voluntary and data collection was audio-taped. Focus groups lasted 78 min 06 s (range: 01 h 09 min 11 s–01 h 25 min 03 s), interviews with supervisors 46 min 19 s (range: 30 min 19 s–01 h 11 min 00 s) and expert interviews 42 min 55 s (range: 29 min 24 s–1 h 05 min 31 s) on average. No repeat interviews or focus groups were performed. Participants neither received their transcripts for commenting or modification purposes nor feedback on the findings.

### 2.4. Data Analysis and Reporting

Audio data were transcribed verbatim (application of the transcription rules of Kuckartz [35,39]) and anonymized. The transcribed supervisor and expert interviews were checked by A.-C.K. and transcribed focus groups by J.C.L. For all qualitative data, the software MAXQDA Analytics Pro (version 12, VERBI GmbH, Berlin, Germany, 2016) was used for encoding. After encoding the first transcript, two researchers (A.-C.K. and I.E. or J.C.L., respectively) discussed the results and developed sub-categories from its text segments. The following transcripts were encoded by one person. Possible uncertainties in terms of text segments or sub-categories were discussed in project meetings until consensus was reached. Data analysis of all formats was conducted using Mayring’s qualitative content analysis [40]. Main categories were developed in a deductive way according to the proposed theoretical framework of the study and sub-categories in an inductive way without the involvement of participants. The interviewees’ quotes were translated into English to exemplify the results. The COREQ-Checklist (consolidated criteria for reporting qualitative research) was applied to report the results (displayed in the Supplementary Materials [29]).

### 2.5. Ethical Considerations

All participants gave written informed consent for data collection. As a supplement, a handout was prepared in plain language for employees with disabilities summarizing the main points. Information on data protection and analysis allowing no references to personal or company-related data were provided in writing and verbally. The study was based on the Declaration of Helsinki. The Ethics Committee of the University Medical Centre Hamburg-Eppendorf, Germany, approved the study beforehand (LPEK-0051).

## 3. Results

### 3.1. Participant Characteristics

Table 4 displays gender, age and work experience of employees, supervisors and experts. Employees and supervisors were engaged in food and beverage service activities and services to buildings and landscape activities. Supervisors also represented further sectors, such as printing and reproduction of recorded media, office administrative, office support and other business support activities, or the wholesale of bicycles and their parts and supplies (traced back to the statistical classification of economic activities in the European Community [41]). Experts were employed in various operational areas, such as superordinate organizations and associations that support the interests of people with disabilities at work, social insurance agencies or governmental institutions. In addition to their engagement in organizations and associations, some experts were also in charge of a subordinated social firm. Likewise, experts for WHP from workshops were included in order to benefit from their experience, despite the limited transferability to the general labor market.

### 3.2. Offers for WHP

#### 3.2.1. Sport Offers

Supervisors described gym discounts, swimming offers, back trainings and that individual colleagues campaigned for fitness programs. No information could be provided on how many colleagues took advantage of these offers. Additionally, gymnastics or various exercises with employees were offered every morning in a company. Sport activities for sheltered workshop employees (holding outsourced jobs in social firms) were also offered by their foundation. An initial defensive attitude among employees and supervisors towards sports offers was reported, which turned into the opposite after participation. For some supervisors, it was impossible to take advantage of offers during working hours, but they encouraged employees to participate.

*“There was once an offer that we […] did some kind of gymnastics […] but in the morning at eight or so, first I was totally defensive against it, because I thought, I can’t let myself be torn out of my work, so I found that totally disturbing at that moment, because I started working at six, then I went to the gymnastics at eight and so out of the daily work routine and then back again afterwards […] I think we did it once a week, I still thought it was good, it did me a lot of good, I have to say, and I also noticed that such a break also works, so that was actually quite an interesting thing in my head, because before I thought it was impossible and then it worked” (Supervisor #5, male).*

The experts complemented various other offers for WHP by social firms themselves, associations or cooperation with providers, such as swimming, assertiveness and self-defense training, yoga classes, running workouts, soccer teams, Zumba groups, gymnastics classes, combat sports offers, climbing, boxing workouts, gym discounts or fitness trainings. Other offers included bike leases initiated by employees. Initially, trails were partly used to test certain offers. As a result, positive outcomes were underlined by experts, including an improved health (literacy), weight loss and regular exercise. These offers were also described as team-building or communication-enhancing approaches that were associated with enjoyment and fun, in which employees learned about their needs.

*“So we support sport, athletic activities […] That you give support and also financial support for fitness and sports” (Expert #11, male).*

Participation in various running and tournament formats was organized for people with disabilities, such as soccer tournaments, Special Olympics, running competitions or company sport groups. These formats provided an incentive to stay active regularly and promoted employees’ identity and pride. In some cases, offers were not labeled as WHP.

*“We also once had such a running training group that one says, we make a group that now, there is this [name of a run] […] where every man, every woman can participate, that one says we form a company group and prepare ourselves for it […] I think these are incredibly effective, incredibly positive approaches, which also, in my observation, bring something to the people, including the disabled employees” (Expert #13, male).*

#### 3.2.2. Nutrition

Supervisors described various nutrition-related interventions, such as efforts by the management to offer whole-grain bread, vegetables and fruits for breakfast; the provision of balanced meals; the organization of cooking courses on various topics and joint market visits; as well as support by social education workers. In addition, individual supervisors provided consultations on the subject of nutrition (without support by the social firm).

*“We also offer courses […] last year I had one, it goes a week, six hours each […] once to know what vegetables and salads the people know at all, because most know only an iceberg lettuce as a salad approximately, that’s it. Then I went to the market with them, looked at the things with them […] They had to choose a salad they would like to make, they actually had to pick out all the ingredients themselves […] for four people and calculate what they might spend on it […] that all worked out relatively well, it was fun, of course it’s exhausting, you have to come up with something for six hours a day, but they all participated well, it was fun for them” (Supervisor #7, female).*

On the subject of nutrition, the experts presented various approaches, such as breakfast improvements. Other suggestions from sheltered workshops included integrative cooking or nutrition courses for people who have voluntarily decided to lose weight.

*“We are still planning a nutrition course that is a bit more narrowly focused for people who have voluntarily decided to lose weight, which is then monitored by the medical service, with regular weighting and individual counseling” (Expert #3, female).*

#### 3.2.3. Relaxation Offers

In one focus group, employees reported that a colleague trained as a professional massage therapist provides massages for colleagues in the team:

*“She does it very well actually, because I have back pain […] and whenever she did it, it’s always like I, I think a few weeks sometimes or a phase even a month I had no pain” (Employee #1, female).*

Supervisors reported offers for relaxation in the social firm either through the supervisors own initiative or likewise through trained colleagues offering massages.

*“A few times, so every few weeks I have then brought my, my sound bowl and then said, now we meditate two or three minutes, so we can see how long three minutes are. Or I turn on mediation music for three minutes or four minutes, so that they can calm down” (Supervisor #4, female).*

Experts supplemented experiences with offers for stress management, yoga during breaks, relaxation weekends or again massage offers.

*“We offer relaxation courses. They do stress management, yoga during the break or something like that, which is then also available to everyone” (Expert #10, male).*

#### 3.2.4. Smoking Cessation

Supervisors highlighted the provision of smoking cessation offers.

*“We have also been able to stop one or two people from smoking” (Supervisor #12, female).*

Experts were in line when referring to smoking cessation as part of the company health management for all employees (not only for the social firm of the company).

*“We have various offers as part of the company health management for all our employees that is not only for those in the social firm available, but for all employees. These range from smoking cessation courses to sports activities […] free of charge” (Expert #16, male).*

#### 3.2.5. Cooperation with External Organizations/Health Actions

Furthermore, the implementation of health checks in some social firms was mentioned and supervisors promoted preventive measures of health insurances.

*“Health insurances are obligated to offer these preventive measures, you can make that via certain providers […] I communicate that to my coworkers […] some write it down by themselves and are interested and others say that they do not need it” (Supervisor #8, male).*

The experts added various examinations, such as colon cancer screenings, flu vaccinations and programs providing incentives by health insurances.

*“What worked well was the start of the program with [name of the health insurance] […] For them it was an incentive, they could get up to 600 euros paid out in cash through this three-year program […] So I think in the end we had a pretty high quota of those who participated in the program and it was very well received […] For membership in a sports club, membership in a fitness center, participation in a course called ‘Finally a Non-Smoker’” (Expert #16, male).*

Additional suggestions from sheltered workshops included health actions once a year, which were readily accepted and created awareness, but quickly faded out, e.g., in collaboration with health insurances and on the topic of stress, relaxation training, heavy lifting, nutritional advice, hygiene training or with other topics for groups with different stations. A low-sugar year or a healthy month were also mentioned. Further experts reported on other programs, such as a curriculum on health.

*“We then called the whole thing the low-sugar year […] and the specialist service for training and development visualized this with pictograms, with posters, with small films, with training courses and sports offers” (Expert #3, female).*

#### 3.2.6. Training and Education Offers

Employees rated their participation in communication or music seminars as positive.

*“And I had done this ‘CoCoCo’ seminar, that is, communication, conflict management, cooperation. And then we also did a lot together. Role-playing and writing things down together […] for more teamwork and so on, right?” (Employee #2, female).*

Supervisors also informed about trainings and seminars in social firms via the employer’s liability insurance association or superordinate institutions, on occupational safety and prevention, communication or disability-related topics (e.g., sign language, pedagogical skills or mental health conditions), as well as excursions or creative offers for employees (music groups or pottery courses). A reflection after four weeks was possible in some cases. Supervisors were also able to select seminars for employees on various topics, such as communication, work–life balance or mobbing. Likewise, they were able to educate themselves further with several training formats (e.g., safe driving or agile management). Company doctors also informed about skin care or solvents.

*“My impression is that [name of the superordinate institution] already covers a lot of ground there, and of course we also have the opportunity via [name of two providers] to send people there so that they can organize themselves better or resolve conflict discussions better at the end of the day. There are also such training courses” (Supervisor #3, male).*

Experts were in line and reported on a range of training courses for employees and supervisors (also purchased externally). For instance, training and consulting opportunities for supervisors on how to deal with challenging employees, mental health conditions and disabilities were mentioned, since supervisors usually do not have any specialized education in the field. In the same vein, case supervision, network meetings for training officers or internal mentoring programs were offered.

*“For the supervisors there is an opportunity […] for case supervision, where you can bring up cases from your area, which you had with the person with disabilities and to play through this case supervision […] in order to have clarity for yourself, how did I stand there, did I react sensibly? Because it is often the case that some people do not take it to heart or take it to heart very little and continue to do their job […] and others take it to heart far too much and could then become mentally ill at some point, so you have to make sure that you offer support” (Expert #12, male).*

For employees with disabilities, offers from the provider of their institution or other counseling services were reported, along with aptitude tests and training for the job as well as training on communication and stress management.

*“I think it is important that people are enabled to communicate appropriately and that they are able to cope with stress, i.e., relaxation techniques or stress management, and that they practice targeted intervention” (Expert #13, male).*

Additionally, annual training catalogs for employees with focused topics and health trainings as well as tandem events in which people with and without disabilities attend workshops together were suggested by experts from sheltered workshops.

*“Further education and training courses are a regular part of the training catalog, in any case, and are called self-assertion […] Being able to say no […] cognitive skills to train, to find a way for themselves, also on the subject of health […] but not only.” (Expert #3, female).*

The latest developments in the context of training courses due to the pandemic showed that face-to-face workshops can also be implemented as video conferences and that full-day events can sometimes be reduced to smaller half-day events.

*“We are making such experiences that we have now moved away from face-to-face workshops, which are mainly aimed at supervisors or trainers […] and are now doing a lot in video conferencing or in the online process and we are making good experiences […] which, of course, already saves time, so it works quite well at first, you can see that we are now expanding it” (Expert #13, male).*

#### 3.2.7. Other Health-Related Offers

Other offers included first aid courses, violence prevention, excursions or theater groups.

*“We have had mandatory courses in violence prevention for all employees, also as part of health management […] From these further training courses, new impulses always arise […] We do this with a professional provider who carries out this training […] We have trained our own internal trainers, who then provide internal training for the employees. And that starts with dealing with difficult situations or with escalating behavior and goes all the way down to conversational skills, to non-verbal communication, and so on. That’s just this training. All of our employees do this, no matter in which area, no matter in which function” (Expert #16, male).*

Results on offers and needs for improvement of WHP are summarized in Table 5.

### 3.3. Needs for Improvement on WHP

#### 3.3.1. Sport Offers

Several needs for improvement on sport offers were reported by employees and supervisors. For instance, gym discounts, special conditions for use of swimming pools, a punching bag to vent on or more back trainings were requested. To enhance participation, financial incentives were demanded, e.g., when buying a bicycle or taking part in running offers. An early end of the work day was suggested as a reward by supervisors.

*“So I thought to myself, is it somehow feasible that we maybe go to a fitness center? Is that possible? Or whether our company supports this in some way […] So that we have the opportunity to do sports or something like that after work” (Employee #11, male).*

*“As far as I know, there are companies that pay for a bike. Or rent a bike and then […] one inevitably rides a bike more often. Something with sport that motivates you” (Employee #6, male).*

#### 3.3.2. Nutrition

Some employees expressed their wish for cooking healthy food together in the team at work and also to receive support in implementing a healthy diet.

*“Cooking as a team […] We already do that in private. That’s why I suggested […] something healthy, because [name of colleague] […] can’t always implement it properly” (Employee #1, female).*

Other topics for improvement concerned nutritional counseling, e.g., through external consulting or wishes to be provided with more fruits at breakfast.

*“But improvement in terms of health, maybe at breakfast, maybe a bit of fruit or something, but not really that big. So smaller things I would rather, yes” (Supervisors #2, male).*

#### 3.3.3. Relaxation Offers

Additionally, supervisors’ own mindfulness was stated as a topic that needed to be improved further, such as paying more attention to their own breaks, finding strategies to reduce stress at work or detaching from work. Likewise, on-site offers for relaxation and mindfulness as individual or group offers, e.g., Pilates or yoga once a week, were stated by supervisors. The need for courses on stress management for employees in plain language was mentioned as well, since existing offers were described as difficult to understand.

*“I would find it nice once a week or so […] relaxation or Pilates or yoga training on site” (Supervisor #13, female).*

#### 3.3.4. Cooperation with External Organizations/Health Actions

Collaborations with external organizations offering trainings or projects were suggested, since certain topics can be addressed easier in front of external persons.

*“External people who then also hold a training session, who then also […] stimulate an idea to change something and […] that you also start a project or something together, that you maybe even get a small bonus in some form, whatever” (Supervisor #11, male).*

Experts highlighted the need for guidance to establish contacts with health insurances, e.g., for providing support for smaller companies. Other experts stated the need for free consultation offers and contact persons (e.g., at relevant institutions or where social workers could be reached).

*“So if you develop such a […] sample procedure, such a, such a kind of slide, how to get the health insurance contributions and that you work better with the health insurances for such companies, I think that would be a really good help” (Expert #12, male).*

#### 3.3.5. Training and Education Offers

One employee expressed his need for support in dealing with workplace stressors as he currently does not feel that he can talk about workplace stress.

*“For me, I would like to feel more inside myself when something becomes stressful or particularly stressful or unbearably stressful, that I somehow put on the brakes and find the solution for myself. Or that maybe someone else, who is well trained, finds the solution for me, if I don’t see it […] At the moment at work, I think no [I can’t discuss this well at work]” (Employee #6, male).*

Additional wishes of supervisors included the need for further trainings and the development to more responsible tasks. Other supervisors would like to see more advertising for seminars on dealing with mental illnesses.

*“That we get more technical training for the employees […] that is still developable, very much developable […] And this health thing, so that, that takes place […] very rarely.” (Supervisor #9, male).*

Experts were in line informing about needs for improvement on trainings for employees without disabilities referring to diagnosis and dealing with disabilities, communication training and conflict management as well as training on how to deal with job demands for disabled employees. Furthermore, a need for offers for people with more severe disabilities as well as visual or hearing impairments was stated.

*“Knowledge of diagnosis, further training in dealing with the disability and the specific challenges of the disability would sometimes be quite helpful, so I think some people could benefit from it, if there were appropriate help, support and further training opportunities” (Expert #14, male).*

Concerning the conceptualization of seminars for WHP, there was a desire for smaller units of two hours and a more in-house-oriented approach, in which offers could be made on standardized topics in smaller groups (of 3–8 people), to facilitate the integration into the workday. Additionally, experts explained that trainings with both known and unknown people could be useful. Since the companies had to take care of finding a provider, a topic needs survey or a catalog with questions about the interest and conditions was suggested. However, a need for speakers, company doctors and trainers who are able to present in plain language was mentioned.

*“I think that with smaller units, let’s say with two-hour courses, you could also make a more in-house-oriented offer […] you would achieve more […] they can be quite standardized topics […] I do not think about individual support, so rather group offers where […] three to eight people or six people can participate and then we deal with certain topics there. I could imagine that well, because that is […] integrable in a normal working day” (Expert #13, male).*

### 3.4. Challenges in the Implementation of WHP

#### 3.4.1. Low Take-Up of Offers and Lacking Interest in WHP

Several supervisors described that the same employees take part in offers for WHP, while others want to rest or continue working without seeing any advantages. Other supervisors provided an estimation about interested employees or informed that most ones prefer to learn on the job or on technical topics rather than in seminars on WHP. Therefore, offers were partly discontinued due to a low number of participants.

*“That is with our employees, I would say maybe one-tenth are open to it. Yes, I don’t think that the majority of them say, ‘Oh great, here comes another training course on health prevention, great.’ It’s more like, ‘Yes, this is a compulsory event, we have to take part now, well, hopefully it will soon be over’. So there, that’s more my experience” (Supervisor #8, male).*

Employees’ initial skepticism was also stated by supervisors when implementing measures of WHP, such as changes in diets. In the same vein, individual supervisors showed little interest in WHP.

*“I have the feeling that it is received negatively, because of the changes, I think many are simply not open to new things, then it is first questioned, ‘Yes, why do we have to move there now twenty minutes or why do we today get no spread, but must now eat an apple and a banana?’. But after a week, after two weeks, it was a matter of course and no one questioned it anymore and it was explained to them that it is healthier, that it is for their health, and after that no one questioned it anymore, it was accepted directly” (Supervisor #6, male).*

Experts added challenges when gaining acceptance on WHP offers among the workforce due to a lack of understanding and low levels of interest, especially on nutrition or sport offers despite a presentation of objectives in meetings and the provision of working time and rooms. This was traced back due to the below reasons.

*“Either they are not motivated to take advantage of sufficient external offers, but there is also such a blocking capability against it, which is part of the uncertainty, and then somehow in the free time, so in one’s own free time that [WHP offers] takes place and yes, so convenience, which somehow impedes that” (Expert #13, male).*

In addition, for some sectors the participation in trainings was emphasized as a challenge.

*“Extremely beneficial were the seminars at [name of the provider], unfortunately not many have participated […] That is hotel life, yes? We are always short-staffed, we always have a lot of work and we just have work that needs to be done now […] Many hotel managers find it very difficult to structure and organize so that the vacations are planned in time […] Plan vacations, give security for the employees, as far as possible in the duty rosters” (Expert #11, male).*

#### 3.4.2. Lacking Resources for WHP

The employees’ or companies’ lack of financial resources were described as a challenge, e.g., for excursions or for gym memberships according to supervisors. Other challenges included that perks for gym classes from the provider were not usable for employees.

*“We can now just say, ‘Let’s go as a team, once a week we go swimming’ […] or we do other things, that all costs money and the employees don’t have the money to do that, because they don’t have much money to do such things, and I don’t think that they can afford a gym now. But of course we can’t pay the gym fee for [number of employees] either” (Supervisor #1, female).*

A lack of resources concerning personal, time and money was also stated by experts, especially for smaller companies facing strong time, economic and production pressures affecting the whole workforce. Furthermore, less savings, higher employee costs and tightly calculated profit margins were underlined, resulting in problems establishing suitable structures for WHP. Complaints were described concerning the staffing levels, meaning they can only deal with core business and instructional activities accompanied by many unfilled positions, high workloads and the employees’ need for support. Limits of support and advice were also mentioned when referring to the employees’ needs, whereby social education workers were demanded.

*“We’re in such a difficult position anyway because we have higher personnel costs here, higher employee costs than other businesses. We have to somehow absorb that and somehow cope with it and then […] even more tasks and duties. Then it becomes difficult for an inclusive business to be able to cope with this economically” (Expert #11, male).*

In workshop settings, it seems to be easier to offer services within working hours, since social firms are exposed to greater economic tension on the general labor market. Some experts assumed an additional economic expenditure, e.g., when staff has to take care of WHP. In the same vein, unknown support possibilities were described due to the fact that no one takes care of the topic full-time.

*“If you say, we provide now additional offers, which must be dated, then nevertheless as a rule outside of the regular core working hours, because one otherwise has […] a restaurant which is open, or one has somehow certain things to deliver, so one does not get finished in the working time […] it is difficult, if one makes that now on a voluntary principle, to motivate the people to participate […] with large enterprises it is a discussion with the works council, with, with smaller ones is likewise priority setting, do I want to go home fast or do I still take part and so and I find that somehow, so there the workshops have it easier, they put it simply in the middle, but that does not apply to social firms in such a way […] That’s just simply a field of tension” (Expert #13, male).*

#### 3.4.3. Lack of Structure in WHP

Partly as a result of the previous mentioned missing resources, a lack of structured processes and knowledge and large differences in the degree of implementation of WHP were observed in social firms (interventions usually about team building, compatibility of work and family domains or the use of company cars for private reasons). In general, structures and processes should be created and adapted to the employees’ needs.

*“It was more something that actually came from the employees and was more about team building or somehow, yes, strengthening a sense of community […] But this is actually not something that would have been based on systematic occupational health management” (Expert #8, female).*

Other experts mentioned that the topic of WHP was often described as very complex without providing suitable definitions and that the setting of social firms included a wide range of business activities (e.g., crafts vs. more science-oriented companies).

*“Yes, there were many ideas, where one always asked oneself, ‘What is health promotion? So where does it start, where does it end? Or what is also everyday life, what also includes leisure activities?’ So you could develop a lot of things, but that takes time. And then you also have to look at who finances what? So I would say that the water dispenser for everyone is not for me, from my point of view, not the greatest health promotion. But you can also run it under that. But it’s not the big thing” (Expert #17, male).*

In general, it was reported that few social firms have works councils, inclusion officers or representatives for its employees, although they already meet the requirements.

*“Who is the inclusion officer in the inclusion company? So it’s not just the representative body for the severely disabled and the works council, but also: Who is the inclusion officer? They are appointed, they exist, but they don’t show up very often for training courses” (Expert #17, male).*

#### 3.4.4. Compatibility of Offers with Work Time and Organization

Organizational challenges when implementing measures for WHP included work-time-related difficulties, e.g., due to different working hours or break times of employees or a lacking willingness to participate after work or in the morning. For some supervisors themselves, participation during working hours was also not possible. On the other hand, free spatial capacities were mentioned, especially in the afternoon.

*“I think that it could come to time difficulties […] because there are different working hours and breaks are limited, so to reconcile, would probably also be difficult with food and training and whether people would still want to stay after work is questionable” (Supervisor #13, female).*

#### 3.4.5. Challenges Related to Available Training Offers

Different challenges were stated based on experts’ experiences concerning the provision of trainings, including a lack of suitable training offers from the perspective of the social firms focusing on workshops for people with disabilities in the first place.

*“The relevance of occupational health and safety is high […] and there are many health risks on both sides. However, they also say that they have no use for certain things that we offer, that we call industry-specific, because they are very much geared to this area of vocational rehabilitation and workshops for people with disabilities […] and therefore you have to take a closer look at this target group of social firms” (Expert #6, female).*

Difficulties regarding language and comprehensibility were also raised by experts, when finding speakers offering plain language and intercultural approaches.

*“It’s actually just really difficult to find speakers or people who can do it in such a, how should I say, plain language and multicultural, that’s what it has to be, so not only different nationalities, but also different cultures of people, so disabled, non-disabled, women, men? […] So there is simply a kind of database missing” (Expert #7, female).*

Furthermore, social firms were partly not familiar with available offers, the communication process with employees was described as difficult or people with hearing impairments were partly left out.

*“The training process for colleagues in recent years was really difficult, that you could communicate to people, we have training, we take care of formats […] and then you realize […] you […] have left the people with, with hearing impairment, so to speak, a little bit outside. Because that is again another, an independent group, which then again requires other necessities in the, in the further training planning” (Expert #12, male).*

#### 3.4.6. Challenges Related to the Management or Supervisors

Experts stated difficulties of managers identifying themselves with and in turn supporting the implementation of measures for WHP and providing resources, especially for smaller companies. The qualification of the management was explained as a reason, whether a managing director is a business economist or a social education worker.

*“And then it also depends on what qualifications the managing directors have. Well, I can see that there are big differences in whether a managing director is really a business person, i.e., a business economist, or whether he or she is a social education worker or educator […] So from the occupational group of social education workers, psychologists, so everything that goes into the interpersonal, ethical area and into the helping area, but at least 50% or more business administration is demanded. And that’s often not really compatible, because you haven’t learned that either. And it’s the same with someone who is a business economist, where at least 50% empathy is also required, which is also often not compatible” (Expert #17, male).*

A further challenge was depicted when recruiting supervisors and building new inclusion projects as they need time to understand how to prepare work processes and deal with disabled employees. For instance, in the hotel or gastronomy sector, finding non-disabled skilled staff was emphasized as the jobs were characterized as demanding.

*“And what you then hire as non-disabled personnel must be compatible and fit […] a quite normally trained cook, a kitchen is always also an explosion barrel, there are quite different tones. So you have to find a cook who also has empathy for this group of people […] but finding these people is even more difficult than simply hiring a chef. So you have a big difficulty overall with filling the jobs already. And you can’t pay more than above the pay scale […] With totally different working conditions” (Expert #17, male).*

#### 3.4.7. Challenges Related to the Employees’ Needs and Capacities

Supervisors added employees’ receptivity as a challenge when designing measures for WHP or when employees attain training offers. For this reason, supervisors reflected that the individual needs of employees make it difficult to standardize measures of WHP.

*“[Name of the provider] does a lot there and we have already sent people there and they [employees] have simply, in part, simply not understood, or cognitively cannot grasp […] I don’t know why, whether the seminars then simply had an approach too high, also linguistically or so, that is just of course the great difficulty, to then screw down to the level, people are sitting there, and to make it understandable for them” (Supervisor #5, male).*

Experts supplemented that individual needs of employees should be considered also according to different disability-related patterns without applying a generalization of offers (e.g., for sensory disabilities and their environment perception, mental health conditions and the confrontation with the range of job demands, hearing-impaired employees and the provision of language interpreters). Further challenges were stated as persistent symptoms, medications or limited abilities to organize.

*“I would say that what is still a building site is to find out exactly for people with mental disabilities or mental impairments, what exactly do they need? Or what exactly do they want? I think our project team or we as a company are always looking for new ideas, for examples of best practice, and we try to look left and right to see what other organizations are doing. What offers are they making for employees? I think that’s a challenge. That you always have to take a closer look […] We also employ hearing-impaired people. With hearing-impaired people […] it is much more important that communication works. So, that in case of doubt, I have organized sign language interpreters and people can participate in their communication, so to speak” (Expert #16, male).*

As an aggravating factor, experts added that many companies faced an aging workforce resulting in a perception of declining performance and questions whether financial compensation was sufficient. Furthermore, many employees went back to a sheltered workshop after 15 years because due to more strenuous working conditions. Other challenges came across due to higher (long-term) sick leave rates in social firms or due to the employees background (they often lived alone or were unemployed for a long time).

*“We also know of some companies that say it is difficult […] because we now have aging workforces, there are many companies that have now passed the 30-year mark since they were founded and the employees have generally grown old with them. And then, of course, there is also the perception of declining performance and ‘What am I going to do now? Are my disadvantage compensations actually still sufficient?’ […] In order to be able to support the employees until retirement, so to speak, so you already get that, they are just the challenges, to look, dealing with aging workforces, yes, health promotion” (Expert #2, female).*

#### 3.4.8. Other Challenges

Other challenges included unsuitable survey instruments, lacking appreciation in the hotel sector, achieving long-lasting changes in the employees’ behaviors, voting rights in the company when discussing further training or education offers or people with disabilities who hide how challenging a task is at work.

*“And there is also the difficulty that actually, this should work without any particular difficulty and many people with disabilities, which I have met so, of course try in the moment, where it is judged, to show that they can already do it very well, so, also, and do not talk about how exhausting it is partly maybe […] they would then sometimes be satisfied with a semi good solution, simply in order not to be considered difficult, so to speak” (Expert #8, female).*

Contrary to the legal obligations of social firms, individual experts raised the question of boundaries between the work and private lives of employees, as well as responsibilities of care facilities regarding health promotion. Challenges with social associations were also mentioned.

*“The biggest challenge for social associations, in my mind, is the business mindset. Yes? The thinking of the social associations is, of course, very social and very focused on caring for the employees, and often the business management, or let’s say the business management necessity, often gets lost in the process. Many social associations are strong enough to absorb this crosswise. This is usually not presented to the outside world, yes? You can also justifiably say that they say, ‘We have or earn so much money with our activities that we can finance these inclusive jobs’. That’s a justified argument, I think, but if I want to say that a social firm must be self-supporting and self-financing, then I must also give the business management sufficient space” (Expert #11, male).*

Table 6 summarizes challenges in the implementation of WHP in social firms.

## 4. Discussion

The present study was one of the first examining offers for WHP, needs for improvement and associated challenges in the context of German social firms providing employment for people with different disabilities on the general labor market. Based on the displayed research questions and theoretical framework, employees, supervisors and experts reported several WHP approaches, including sport offers, nutrition, relaxation, smoking cessation, cooperation with external organizations and training or education offers. Needs for improvement were highlighted, including additional sport offers or gym discounts, financial incentives, support for a healthy diet or cooking with the team as well as offers for relaxation or education. Likewise, further cooperation with external organizations were requested. As stated by supervisors and experts, challenges referred to a low take-up of offers; a lack of resources, structures or management support; compatibility of offers with work time and organization; challenges with available trainings or the consideration of individual needs and capacities.

### 4.1. Offers and Needs for Improvement for WHP

The results indicated a broad range of individual offers for WHP and associated needs for improvement, including the domains of sport, nutrition, relaxation and smoking cessation. Beforehand, no WHP interventions could be identified in the setting of social firms within the current state of research [12]. However, first insights were provided by a recent report on social firms in Germany, indicating that 71 percent of the participating 93 social firms provided WHP by reducing workloads or anchoring health promotion in the corporate culture (50.5%). However, behavioral-related offers were provided less frequently [42]. In addition, around 80 percent stated that they have not developed an individual WHP concept in comparison to already existing ones from health insurances. Other social firms stated that local offers are lacking; that the use of fitness studios, yoga, swimming courses or back training were viewed as not comprehensible enough; or that existing offers were less adapted to the individual situation of the company and the specifics and requirements of the workforce [42]. All three aspects were found in the present results, including existing cooperation with social insurance agencies or other institutions, needs for improvement on local offers like gym discounts and unsuitable interventions concerning plain language or distinctions to those for workshops. Therefore, further research and company-specific approaches are needed. Likewise, the use of reward systems was stated as a need for improvement, which was supported by Robroek et al. [43], who found higher participation rates when incentives were provided.

Furthermore, different training and education offers were highlighted in the present results, accompanied by needs for improvement in terms of advertising, group sizes or language levels. High demands for further training when employees are supported in the transition to other companies on the general labor market, when working in contact with customers or when technical qualifications presented an advantage for the company were also found within the previously depicted report [42]. Content-related needs for further training were in line with the presented results in three main areas, including professional qualifications depending on the activity (e.g., further training in cleaning and hygiene), in cross-occupational topics (e.g., social behavior or self-organization) and in dealing with employees (e.g., dealing with mental illness). One-third of training courses were aimed at health promotion [42]. The results were in line with Canadian findings, stating that supervisors partly received training about mental health conditions in social firms [44], especially in view of the fact that individual needs of employees with mental health conditions occur less visible [45].

Due to a lack of interventional research within the setting of social firms, further experiences from related areas like workshops could also be considered; however, these are only transferable to a limited extent due to the heterogeneous group of employees and the framework conditions on the general labor market. Hublet et al. [20] reported on WHP in sheltered and social workshops in Belgium, which implemented interventions on the topics of alcohol intake (58.5%), nutrition (50%), mental health (37.8%), smoking cessation (36.6%) and sport-related offers (28%). Behavioral-related offers and education in groups were present in 43.1% and 31.5% of companies, respectively [20]. Other evaluations of single interventions in workshops were evaluated as predominantly positive, impacting the employees’ health status and literacy; decreasing mental stress at work; reduced depressiveness and anxiety [16]; or improving self-organization, social interactions, performance, work processes and attention [19]. In addition, inquiry-based learning helped to develop a more complex understanding of health and fostered social skills and self-confidence [17]. Likewise, a reduction in health risk factors to the employees was observed after cardiometabolic training [18]. However, long-term evaluations after four months also showed opposite effects, such as increased unjustified worrying, although with positive effects on quality of life [15].

### 4.2. Challenges in the Implementation of WHP

Offers and needs for improvement were accompanied by several challenges, such as a low take-up of offers for WHP. Supervisors’ estimations on participation rates of WHP reflected the tendencies from previous research, which were typically under 50% [43]. High attrition rates, a lack of attendance and motivation and aggravated recruitment processes were also factors in the findings of Naaldenberg et al. on health promotion interventions for people with intellectual disabilities [14]. In this context, a prevention dilemma can be discussed, whereby groups with higher intervention needs tend to have lower acceptance and attendance rates compared to those with lower needs due to their higher social status and better health outcomes. The latter recipients, however, are much more likely to take advantage of WHP offers, which also reinforces health inequalities on a structural level [46]. Further challenges were depicted, including individual needs and capacities of employees or the use and offering of plain language in trainings. Research from related fields are consistent with the present results, showing that interventions and information materials were not altered for people with disabilities, impeding a low-threshold attainability [14,20,47].

Challenges on an organizational level referred to a lack of resources, including personnel, time and financial constraints, which were also found for other companies on the general labor market [14,48,49]. The compatibility of offers with work time, the implementation of WHP for different work sites and spatial capacities were also stated as barriers by supervisors and experts. In general, employees’ workloads and schedules, including overtime, part-time work, irregular work schedules and time pressure as well as limited flexibility to leave the workplace, willingness to provide time to participate and the number of company locations, were all common challenges in the literature [14,48,49]. Therefore, sufficient time should be made available when offering WHP interventions, which should be compatible with the time schedule and communication channels, while already existing meetings should also be used [49].

As an aggravating factor, supervisors and experts mentioned challenges during the implementation of WHP due to economic pressure when maintaining daily business operations, which especially applied for smaller social firms. A recent analysis of working conditions in social firms was in line, highlighting conflicts between social and economic objectives as a job demand of supervisors [26]. However, with regard to supervisors and in line with the present results, challenges in recruiting personnel with suitable social skills were also evident in other studies (about 75% agreed or strongly agreed) [42].

Another factor that was presented and also discussed for other companies on the general labor market was the lack of management support in planning, implementing and evaluating WHP programs [14,48,49,50]. However, Meacham et al. [50] evaluated management perspectives on WHP for employees with intellectual disabilities as a critical factor for the success of an intervention, and in turn on employees’ workforce participation. Wierenga et al. [49] emphasized the company’s awareness of the benefits of WHP and the underlying economic impacts, the improved image when caring about their employees and their perceived responsibility as an employer towards the employees’ health as facilitators when introducing WHP.

Additionally, there were still differences according to company size—the larger the company size, the more care was taken to improve the employees’ health in other studies [51,52]. An often-neglected factor in the current research is the comprehensiveness of WHP interventions in practice. Our results indicated that social firms reported a lack of structure and definition for WHP in social firms. According to Beck et al. [52], only 9% of the analyzed companies implemented a wide-ranging model by offering an analysis along with behavioral-related and structural-related approaches. For this reason, a holistic concept tailored to the needs of small- and medium-sized companies should be promoted. Interventions with several components compared to structural or policy changes only as well as those based on individual behaviors showed increased attendance rates and were proven to be most effective [28,43,53]. However, it was found people with more complex disabilities that they were worse addressed and reached by interventions, which applied especially for people with learning, intellectual or multiple disabilities as well as for those with a migrant background [13].

### 4.3. Strengths and Limitations

When interpreting the presented results, strengths and limitations have to be taken into account. On the one hand, triangulated qualitative methods were chosen to explore findings for an occupational setting for which little research exists on the topic of WHP. Compared to individual interviews, focus groups (taking hygiene and distance regulations into account) promoted familiarity, engagement and facilitated interactions [37]. Due to the latter factor, sign language interpreters would have been available for focus groups but were not used, since participating hearing impaired employees were able to speech-read. On the contrary, telephone interviews increased flexibility for participation for supervisors and experts. For supervisors, the PCI technique was applied to gain insight into their experiences, perceptions and reflections [38]. By including snowball sampling for expert interviews, a homogeneous sample was obtained, which was suitable for examining present research questions in detail [31]. For all participating groups, a diverse age, gender and sector composition was aimed for as well as different types of disability and diverse groups of subordinate employees to provide exploratory insights for the setting of social firms. Furthermore, direct quotes form participants were presented to underline the findings. When analyzing the data, consultations within the research team maintained transparency and accuracy of data. Moreover, the sample size of the interviews can be considered as a strength, indicating the provision of sufficient data to assume saturation in earlier studies [54]. Similarly, focus groups followed the recommended group size (three to ten employees) and amount of groups (three to five focus groups) [35,55].

On the other hand, several limitations were inherent to the study design. Due to the qualitative approach of the study and its objective to provide exploratory in-depth findings, the generalizability of results is limited. In light of the German legislation for social firms, the transferability of results is also restricted for employment opportunities in different countries following varying legal frameworks. Another limitation results from a possible interviewer bias regarding the participants’ response behavior, which could be influenced by social desirability, or a possible selection bias based on the sampling strategy. Likewise, when interviewing supervisors and experts via telephone, social cues and contextual information were missing, but telephone interviews were proven to maintain data quality [56,57,58]. Lastly, the current COVID-19 pandemic and its precautions also impacted the study. To maintain employee health during the data collection, a strict hygiene concept was developed for each focus group, including a maximum number of participants or compliance with hygiene and distance regulations. To gain insight into work-related impacts, recent research underlines the consequences for employees and supervisors in social firms during the ongoing COVID-19 pandemic [59]. Due to the second lockdown in November and December 2020 in Germany, two additional focus groups could not be accomplished following the restriction of social contacts.

### 4.4. Implications for Policy and Practice

Taking the present results into account, several implications for policy and practice can be deduced. First and foremost, interventions should follow a holistic approach of behavioral and structural components [13,14]. Thereby, the companies’ established communication channels or meeting structures could be used to integrate concerned interventions [49]. The employees should be systematically involved in planning processes, from the problem definition to evaluation of WHP interventions [13,14,48,49]. In terms of empowerment, employees should be encouraged to develop their own abilities further and supported in fostering coping strategies [13]. Furthermore, offers should be adapted with regard to the heterogeneity and varying needs of people with disabilities, as well as age, gender and culture [13]. Therefore, employees should be addressed in an adapted way, including literacy levels and the use of plain language [13,60]. The characteristics of the implementer, referring to their skills, knowledge and competences, should also be taken into account [49]. It is also recommended to include multipliers facilitating the sustainability of the interventions [13], to establish partnerships with different providers of WHP and sport activities [14] and to consider financial compensation or other incentives [49]. At the end of an implementation, evaluation and documentation efforts should be made [13,48], including several outcome measures and qualitative approaches, to increase knowledge about the intervention’s strengths and limitations [14].

Social firms are often classified as small- and medium-sized companies [61], which results in various structural disadvantages, such as lower financial and personnel resources compared to larger companies [62]. On the basis of our findings, the question arises as to what extent WHP can be implemented with the available resources. The high relevance of the topic and the multitude of existing individual approaches were identified, which are primarily dependent on the commitment of individual persons. Therefore, it has to be discussed in labor and health policies and pointed out that a comprehensive WHP approach ties up corresponding resources, such as time to participate in measures, money, personal and infrastructure resources [48,49,62]. If the question of missing resources is not clarified on a structural level, it seems to be hardly possible to act beyond individual measures and in a less holistic way on an operational level. Despite a lack of resources, cooperation between companies and regional partners can already be considered by pooling resources while maintaining transparency and commitment between the partners, ongoing networking and exchange, coordination and implementation by service providers as well as technical and financial support by health insurances [62]. The latter should also be made aware of the framework conditions of social firms on the general labor market, since a lack of suitable training offers was described focusing on workshops for people with disabilities in the first place.

Additionally, responsible managing directors were identified as central players. They should become aware of their health-promoting role, as they can encourage employees to adopt health-promoting behavior, establish a suitable management culture and achieve sustainable implementation of WHP through their support [62]. The implementation and functioning of advisory boards are also described as facilitating aspects for WHP [49].

In the present results, it was reported that individual health actions only had little success in the long run. Sayed and Kubalski [62], however, showed that health actions can be used as a starting point and as motivation for implementing further health-related topics in a company to sensitize the employees. Furthermore, interventions should be seen as part of the company’s comprehensive health policy rather than as an independent project. Ideally, health management processes consist of six steps, from the determination of needs (e.g., through working groups, target definitions and financial planning) through to analysis (e.g., by means of surveys and intervention planning) and the implementation, evaluation and consideration of sustainability [62]. Ensuring a holistic approach in addition to WHP as a pillar of occupational health management, high priority should also be given to occupational health and safety, workplace integration management and organizational development and leadership [3].

### 4.5. Implications for Future Research

Several implications for conducting further research should be taken into account when analyzing the topic of WHP for people with disabilities. As a basis, ongoing health-related disparities should be recognized for people with disabilities, including differences in health-promoting behavior, such as physical activity, nutrition, social contacts, medical care, smoking status and limitations in access to services and programs [21,22,63]. People with disabilities were also described as being underrepresented in the current research in terms of their needs and requirements [13], e.g., as a result of challenges in recruitment and retention [64]. Therefore, participation in further research is important, requiring an inclusive research agenda from problem definition to implementation and evaluation [65]. In this context, both qualitative and quantitative methods should accompany interventional and longitudinal studies examining associated experiences, challenges and health-related outcomes, also depending on the type of disability. However, the current research on WHP is characterized by challenges relating to study design, such as in the implementation of control groups and randomization due to time and financial constraints, small sample sizes, a lack of transparency in existing interventions and evaluations and a lack of the sustainability in interventions. Regarding measures and outcomes, the absence of standardized scales for people with disabilities was also discussed, with no control for intervening factors, social desirability or long-term outcomes [13,14]. Diverse samples should be taken into account regarding age, gender, position, type of disability and company size. Synergy effects and networking opportunities among small- and medium-sized social firms could also be a focus of future research. Additionally, different country- and sector-specific factors should be examined.

## 5. Conclusions

These results regarding WHP offers were the first within this research field and provided insights into how WHP has already been implemented in social firms. Beforehand, no WHP interventions could be identified in the setting of social firms within the current state of research, while individual studies and interventions were only available from workshop settings, which are only comparable in a limited way.

On the one hand, experiences from employees, supervisors and experts show that there are already several offers available, such as sport, nutrition and relaxation offers as well as those on smoking cessation, cooperation with external organizations and training and education offers. On the other hand, there are specific needs for improvement, especially with regard to establishing a holistic and sustainable WHP approach. Likewise, the implemented measures were accompanied by some challenges, such as a low take-up of offers; a lack of resources, structure and management support; compatibility with work time and organization; challenges with available trainings or the consideration of individual needs and capacities. Although individual approaches were presented, the issue of missing resources needs to be addressed on a structural level. Likewise, responsible managing directors need to be aware of their leading role in the implementation of WHP and need to be supported by social insurance agencies. Overall, there is a need for further interventional and longitudinal research on WHP offers in social firms.

## Figures and Tables

**Table 1:** Current research on WHP in social firms.

Research on WHP in Social Firms
WHP can keep the workforce healthy, e.g., in terms of eating behavior, physical activity, mental well-being, decreased sickness absences or increased work ability; People with a disability are not yet sufficiently taken into account in current WHP offers or research; Only individual studies or interventions are available on WHP in workshop settings; WHP is necessary, since people with disabilities often rate their subjective state of health and psychological well-being lower, face a higher health burden, are less physically active and have less favorable health behaviors; No studies on WHP offers, needs for improvements or accompanied challenges are available within the setting of social firms.

**Table 2:** Applied inclusion criteria.

Inclusion Criteria Domain	Employees	Supervisors	Experts
Employment status	Employees with a disability in a social firm (§215, Book Nine of the German Social Code (SGB IX))	Supervisor in a social firm (§215, Book Nine of the German Social Code (SGB IX)) with direct contact to disabled employees	Expert in the field of providing health-promoting working conditions for people with disabilities or in the field of WHP in general
Age	At least 18 years old	At least 18 years old	At least 18 years old
Work experience	-	At least half a year	At least three years
Working hours	-	At least 18 h per week	-
Language	German-speaking	German-speaking	German-speaking

**Table 3:** Topics of the interview guideline for employees, supervisors and experts.

Employees	Supervisors	Experts
Experiences/offers for WHP (e.g., “Who or what supports you at work?”)	Experiences/offers for WHP (e.g., “Are there any offers for WHP in your social firm?”)	Experiences/offers for WHP (e.g., “Are there any experiences with WHP in social firms?”)
--	Challenges in the implementation of WHP (e.g., “What barriers do you perceive concerning WHP in your social firm?”)	Challenges in the implementation of WHP (e.g., “What barriers do social firms face when offering WHP?”)
Needs for improvement in WHP (e.g., “What could the company do to make you feel better?”)	Needs for improvement in WHP (e.g., “What needs for improvement do you have in terms of WHP?”)	Needs for improvement in WHP (e.g., “What needs for improvement do social firms have in terms of WHP?”)

**Table 4:** Participant characteristics (*n* = 47).

Variable	Employees (*n* = 14)	Supervisors (*n* = 16)	Experts (*n* = 17)
**Gender**			
Male	7 (50.00%)	11 (68.75%)	11 (64.71%)
Female	7 (50.00%)	5 (31.25%)	6 (35.29%)
**Age**			
18–30	5 (35.14%)	0 (0.00%)	0 (0.00%)
31–40	1 (7.14%)	7 (43.75%)	1 (5.88%)
41–50	6 (42.86%)	3 (18.75%)	2 (11.76%)
Older than 50	2 (14.29%)	6 (37.50%)	14 (82.35%)
**Working experience**			
Less than a year	2 (14.29%)	1 (6.25%)	0 (0.00%)
1–3 years	1 (7.14%)	9 (56.25%)	1 (5.88%)
More than 3 years	11 (78.57%)	6 (37.50%)	16 (94.12%)

**Table 5:** Offers and needs for improvement of WHP according to employees (Em), supervisors (S) and experts (Ex).

Offers for WHP	Needs for Improvement on WHP
**Sport offers**	
Fitness programs, gymnastics, swimming, back trainings, self-defense, yoga classes, running workouts, soccer teams, Zumba, combat sport offers, climbing, boxing or other exercises (S and Ex) Gym discounts and special conditions for use of swimming pools (S and Ex) Running and sports tournaments (Ex) Bike leases (Ex)	Gym discounts or special conditions for use of swimming pools (Em and S) More back training offers (S) Punching bag to vent on (S) Financial incentives or reward systems, e.g., when buying a bicycle or taking part in running offers (Em and S)
**Nutrition**	
Wholegrain bread, vegetables and fruits (S and Ex) Balanced meals (S) Cooking course (S and Ex) Social education worker support (S) Consultation of supervisors for employees (S)	Support for implementing a healthy diet (Em) Cooking with the team (Em) More fruits for breakfast (S) Nutritional counseling through external consulting (S)
**Relaxation offers**	
Massages (Em, S and Ex) Offers for relaxation by supervisors (S) Stress management, yoga during breaks, relaxation weekends (Ex)	Measures to improve own mindfulness (S) On-site offers for relaxation and mindfulness as individual or group offers (S) Stress management courses for employees in plain language (S)
**Smoking cessation**	
Smoking cessation courses (S and Ex)	-
**Cooperation with external organizations/Health actions**	
Health checks/vaccinations (S and Ex) Promotion of health insurance offers (S) Health actions once a year/month (Ex)	Collaborations with external organizations (S) Contacts with health insurances (Ex) Need for contact persons (Ex)
**Training/education offers**	
Trainings/seminars via different institutions (Em, S and Ex) Case supervision, networking, mentoring (Ex) Use of video conferences and half-day events (Ex)	Need for further trainings/development for employees and supervisors (Em, S and Ex) More advertising for seminars (S) In-house offers of two hours, small groups (Ex) Use of a query of interests (Ex) Need for trainers in plain language (Ex)
**Other health-related offers**	
First aid courses (S) Violence prevention (Ex) Excursions or theater groups (Ex)	-

**Table 6:** Challenges in the implementation of WHP according to supervisors (S) and experts (Ex).

Challenges in the Implementation of WHP
Low take-up of offers and lacking interest in WHP (S and Ex) Lack of resources for WHP (S and Ex) Lack of structure in WHP (Ex) Compatibility of offers with work time and organization (S) Challenges related to available training offers (Ex) Challenges related to the management or supervisors (Ex) Challenges related to the employees’ needs and capacities (S and Ex) Other challenges (Ex)

## Data Availability

The datasets analyzed during the current study are not publicly available due to German national data protection regulations but are available from the corresponding author on reasonable request.

## Supplementary Materials

The following supporting information can be downloaded at: https://www.mdpi.com/article/10.3390/ijerph19020959/s1, S1: COREQ Checklist.

## References

[1] Pundt F., Felfe J. (2017). HoL—Health Oriented Leadership, Instrument zur Erfassung Gesundheitsförderlicher Führung.

[2] Rennert D., Kliner K., Richter M., Knieps F., Pfaff H. (2020). Arbeitsunfähigkeit In Mobilität Arbeit Gesundheit—Zahlen, Daten, Fakten mit Gastbeiträgen aus Wissenschaft, Politik und Praxis.

[3] Pfaff H., Zeike S. (2019). Betriebliches Gesundheitsmanagement: Definition, Ziele, Maßnahmen. Controlling im Betrieblichen Gesundheitsmanagement: Das 7-Schritte-Modell.

[4] Schliemann D., Woodside J.V. (2019). The effectiveness of dietary workplace interventions: A systematic review of systematic reviews. Public Health Nutr..

[5] Díaz-Benito V.J., Vanderhaegen F., Barriopedro Moro M.I. (2020). Physical activity and health promotion programs in the workplace: A meta-analysis of effectiveness in European organizations. J. Workplace Behav. Health.

[6] Martin A., Sanderson K., Cocker F. (2009). Meta-analysis of the effects of health promotion intervention in the workplace on depression and anxiety symptoms. Scand. J. Work. Environ. Health.

[7] Kuoppala J., Lamminpää A., Husman P. (2008). Work Health Promotion, Job Well-Being, and Sickness Absences—A Systematic Review and Meta-Analysis. J. Occup. Environ. Med..

[8] Oakman J., Neupane S., Proper K.I., Kinsman N., Nygård C.H. (2018). Workplace interventions to improve work ability: A systematic review and meta-analysis of their effectiveness. Scand. J. Work. Environ. Health.

[9] Statistisches Bundesamt (Destatis) Herz-Kreislauf-Erkrankungen Verursachen die Höchsten Kosten. https://www.destatis.de/DE/Presse/Pressemitteilungen/2017/09/PD17_347_236.html.

[10] Statista Volkswirtschaftliche Produktionsausfallkosten Aufgrund von Arbeitsunfähigkeit in Deutschland nach Diagnosegruppe im Jahr 2019. https://de.statista.com/statistik/daten/studie/869779/umfrage/produktionsausfallkosten-aufgrund-von-arbeitsunfaehigkeit-in-deutschland-nach-diagnose/.

[11] Barthelmes I., Bödeker W., Sörensen J., Kleinlercher K.M., Odoy J. (2019). Wirksamkeit und Nutzen Arbeitsweltbezogener Gesundheitsförderung und Prävention—Zusammenstellung der Wissenschaftlichen Evidenz für den Zeitraum 2012 bis 2018.

[12] Kordsmeyer A.C., Lengen J.C., Kiepe N., Harth V., Mache S. (2020). Working Conditions in Social Firms and Health Promotion Interventions in Relation to Employees’ Health and Work-Related Outcomes-A Scoping Review. Int. J. Environ. Res. Public Health.

[13] Altgeld T., Rothofer P., Vanheiden T., Sädtler T. (2017). Durchführung einer Bestandsaufnahme von Interventionen (Modelle guter Praxis) zur Gesundheitsförderung und Prävention bei Menschen mit Behinderung.

[14] Naaldenberg J., Kuijken N., van Dooren K., van Schrojenstein Lantman de Valk H. (2013). Topics, methods and challenges in health promotion for people with intellectual disabilities: A structured review of literature. Res. Dev. Disabil..

[15] Deforche B., Mommen J., Hublet A., Roover W., Huys N., Clays E., Maes L., Bourdeaudhuij I., Cauwenberg J. (2018). Evaluation of a Brief Intervention for Promoting Mental Health among Employees in Social Enterprises: A Cluster Randomized Controlled Trial. Int. J. Environ. Res. Public Health.

[16] Latocha K., Wieland R. Betriebliche Gesundheitsförderung für psychisch erkrankte Beschäftigte—Interventionsstudie in einer Behindertenwerkstatt. Proceedings of the 59 Arbeitswissenschaftlicher Kongress. Chancen durch Arbeits-, Produktund Systemgestaltung—Zukunftsfähigkeit für Produktions- und Dienstleistungsunternehmen.

[17] Becker K.-P., Burtscher R. (2019). Gemeinsam Forschen—Gemeinsam Lernen. Menschen mit Lernschwierigkeiten in der Partizipativen Gesundheitsforschung.

[18] Kramer C., Bebenek M., Willert S., Kemmler W. (2016). Betriebliche Gesundheitsförderung für Menschen mit geistiger Behinderung. Dtsch. Z. Sportmed..

[19] Milles D., Wiese J. (2018). Gesundheitsförderung in Werkstätten für behinderte Menschen. ASU Arb. Soz. Umw..

[20] Hublet A., Maes L., Mommen J., Deforche B., Bourdeaudhuij I. (2015). Health promotion interventions in social economy companies in Flanders (Belgium). BMC Public Health.

[21] Bundesministerium Für Arbeit Und Soziales (BMAS) (2013). Teilhabebericht der Bundesregierung über die Lebenslagen von Menschen mit Beeinträchtigungen, Teilhabe—Beeinträchtigung—Behinderung.

[22] Bundesministerium Für Arbeit Und Soziales (BMAS) (2016). Zweiter Teilhabebericht der Bundesregierung über die Lebenslagen von Menschen mit Beeinträchtigungen Teilhabe—Beeinträchtigung—Behinderung.

[23] Statistisches Bundesamt (Destatis) Behinderte Menschen. https://www.destatis.de/DE/Themen/Gesellschaft-Umwelt/Gesundheit/Behinderte-Menschen/_inhalt.html.

[24] World Health Organization (WHO) Ottawa Charter for Health Promotion, 1986. https://www.euro.who.int/__data/assets/pdf_file/0004/129532/Ottawa_Charter.pdf.

[25] European Network for Workplace Health Promotion (ENWHP) Luxembourg Declaration on Workplace Health Promotion in the European Union. https://www.enwhp.org/resources/toolip/doc/2018/05/04/luxembourg_declaration.pdf.

[26] Kordsmeyer A.-C., Efimov I., Lengen J.C., Flothow A., Nienhaus A., Harth V., Mache S. (2021). Balancing social and economic factors—Explorative qualitative analysis of working conditions of supervisors in German social firms. J. Occup. Med. Toxicol..

[27] Efimov I., Lengen J.C., Kordsmeyer A.C., Harth V., Stefanie M. (2021). Capturing and analysing the working conditions of employees with disabilities in German social firms using focus groups. BMC Public Health.

[28] Goldgruber J., Ahrens D. (2010). Effectiveness of workplace health promotion and primary prevention interventions: A review. J. Public Health.

[29] Tong A., Sainsbury P., Craig J. (2007). Consolidated criteria for reporting qualitative research (COREQ): A 32-item checklist for interviews and focus groups. J. Healthc. Qual..

[30] Flick U. (2011). Triangulation: Eine Einführung.

[31] Schreier M., Mey G., Mruck K. (2017). Fallauswahl in der qualitativ-psychologischen Forschung. Handbuch Qualitative Forschung in der Psychologie.

[32] Schütz A., Brodersen A. (1972). Der Gut Informierte Bürger. Gesammelte Aufsätze: II Studien zur soziologischen Theorie.

[33] Wassermann S., Niederberger M., Wassermann S. (2015). Das qualitative Experteninterview. Methoden der Experten- und Stakeholdereinbindung in der sozialwissenschaftlichen Forschung.

[34] Meuser M., Nagel U., Pickel S., Pickel G., Lauth H.-J., Jahn D. (2009). Das Experteninterview—konzeptionelle Grundlagen und methodische Anlage. Methoden der Vergleichenden Politik- und Sozialwissenschaft: Neue Entwicklungen und Anwendungen.

[35] Kitzinger J., Barbour R.S., Barbour R.S., Kitzinger J. (1999). Introduction: The challenge and promise of focus groups. Developing Focus Group Research. Politics, Theory and Practice.

[36] Burtscher R., Allweiss T., Perowanowitsch M., Rott E. (2017). Gesundheitsförderung mit Menschen mit Lernschwierigkeiten—Eine Praxishilfe mit Online-Lernmaterialien. Leichter Lernen mit dem Projekt GESUND!.

[37] Schulz M., Schulz M., Mack B., Renn O. (2012). Quick and easy!? Fokusgruppen in der angewandten Sozialwissenschaft. Fokusgruppen in der empirischen Sozialwissenschaft: Von der Konzeption bis zur Auswertung.

[38] Witzel A. (2000). Das problemzentrierte Interview Forum Qual Sozialforsch. Forum Qual. Soc. Res..

[39] Kuckartz U. (2016). Qualitative Inhaltsanalyse: Methoden, Praxis, Computerunterstützung.

[40] Mayring P. (2015). Qualitative Inhaltsanalyse—Grundlagen und Techniken.

[41] Eurostat European Commission (2008). NACE Rev. 2—Statistical Classification of Economic Activities in the European Community.

[42] Sommer J., Meyer S., Gericke T. (2020). Evaluation der Förderung von Inklusionsbetrieben im Rahmen des Programms “Inklusionsinitiative II—AlleImBetrieb” und des Bestehenden Förderinstrumentariums.

[43] Robroek S.J.W., van Lenthe F.J., van Empelen P., Burdorf A. (2009). Determinants of participation in worksite health promotion programmes: A systematic review. Int. J. Behav. Nutr. Phys. Act..

[44] Corbière M., Villotti P., Dewa C.S., Sultan-Taïeb H., Fraccaroli F., Zaniboni S., Durand M.-J., Lecomte T. (2019). Work Accommodations in Canadian Social Firms: Supervisors’ and Workers’ Perspectives. Can. J. Commun Ment. Health.

[45] Villotti P., Corbière M., Fossey E., Fraccaroli F., Lecomte T., Harvey C. (2017). Work Accommodations and Natural Supports for Employees with Severe Mental Illness in Social Businesses: An International Comparison. Community Ment. Health J..

[46] Franzkowiak P. Prävention und Krankheitsprävention. https://leitbegriffe.bzga.de/alphabetisches-verzeichnis/praevention-und-krankheitspraevention/.

[47] Roll A.E. (2018). Health promotion for people with intellectual disabilities—A concept analysis. Scand. J. Caring Sci..

[48] Rojatz D., Merchant A., Nitsch M. (2017). Factors influencing workplace health promotion intervention: A qualitative systematic review. Health Promot. Int..

[49] Wierenga D., Engbers L.H., Van Empelen P., Duijts S., Hildebrandt V.H., Van Mechelen W. (2013). What is actually measured in process evaluations for worksite health promotion programs: A systematic review. BMC Public Health.

[50] Meacham H., Cavanagh J., Bartram T., Pariona-Cabrera P., Shaw A. (2021). Workplace health promotion interventions for Australian workers with intellectual disability. Health Promot. Int..

[51] Schaefer E., Drexler H., Kiesel J. (2016). Workplace Health Promotion in Small, Medium-Sized and Large Enterprises of the Health-Care Sector—Frequency, Reasons for the Company Management to Take Action and Barriers to Implementation. Gesundheitswesen.

[52] Beck D., Lenhardt U., Schmitt B., Sommer S. (2015). Patterns and predictors of workplace health promotion: Cross-sectional findings from a company survey in Germany. BMC Public Health.

[53] Kahn-Marshall J.L., Gallant M.P. (2012). Making healthy behaviors the easy choice for employees: A review of the literature on environmental and policy changes in worksite health promotion. Health Educ. Behav..

[54] Guest G., Bunce A., Johnson L. (2006). How Many Interviews Are Enough?: An Experiment with Data Saturation and Variability. Field Methods.

[55] Morgan D.L. (1997). Focus Groups as Qualitative Research.

[56] Novick G. (2008). Is there a bias against telephone interviews in qualitative research?. Res. Nurs. Health.

[57] Opdenakker R. (2006). Advantages and Disadvantages of Four Interview Techniques in Qualitative Research. Forum Qual Sozialforsch. Forum Qual. Soc. Res.

[58] Block E.S., Erskine L. (2012). Interviewing by Telephone: Specific Considerations, Opportunities, and Challenges. Int. J. Qual Methods.

[59] Kordsmeyer A.-C., Efimov I., Lengen J.C., Harth V., Mache S. (2021). “One of My Basic Necessities of Life Is Work. That’s Just Broken Away.”—Explorative Triangulation of Personal and Work-Related Impacts for Supervisors and Disabled Employees in German Social Firms during the COVID-19 Pandemic. Int. J. Environ. Res. Public Health.

[60] Inauen A., Jenny G.J., Bauer G.F. (2011). Design principles for data- and change-oriented organisational analysis in workplace health promotion. Health Promot. Int..

[61] FAF gGmbH Auswirkungen der Corona-Krise auf IB. https://www.faf-gmbh.de/wp-content/uploads/2020/05/20-0406_Pr%C3%A4sentation_Umfrageergebnisse_Bund_Corona.pdf.

[62] Sayed M., Kubalski S., Pfannstiel M., Mehlich H. (2016). Überwindung betrieblicher Barrieren für ein betriebliches Gesundheitsmanagement in kleinen und mittelständischen Unternehmen. Betriebliches Gesundheitsmanagement.

[63] Rimmer J.H., Rowland J.L. (2008). Health Promotion for People With Disabilities: Implications for Empowering the Person and Promoting Disability-Friendly Environments. Am. J. Lifestyle Med..

[64] Banas J.R., Magasi S., The K., Victorson D.E. (2019). Recruiting and Retaining People With Disabilities for Qualitative Health Research: Challenges and Solutions. Qual. Health Res..

[65] Kitchin R. (2000). The Researched Opinions on Research: Disabled people and disability research. Disabil. Soc..

